# Supplementary material for: Photo-Methionine, Azidohomoalanine and Homopropargylglycine Are Incorporated into Newly Synthesized Proteins at Different Rates and Differentially Affect the Growth and Protein Expression Levels of Auxotrophic and Prototrophic E. coli in Minimal Medium
Source: Int J Mol Sci. 2023 Jul 22;24(14):11779. doi: 10.3390/ijms241411779 (PMC10380393; doi:10.3390/ijms241411779)
Supplement: Supplementary file 1 [file ijms-24-11779-s001.zip › SI.pdf]

Photo-Methionine, Azidohomoalanine and Homopropargylglycine Have Distinct Effect on the Growth of Auxotrophic and Prototrophic *E. coli* in Minimal Medium, Alter Protein Expression Levels and Incorporate into Newly Synthesized Proteins at Different Rates

Supplementary Information

Jecmen T[1,*], Tuzhilkin R[1], Sulc M[1]

[1] Department of Biochemistry, Faculty of Science, Charles University, Albertov 2030, 128 43 Prague 2, Czech Republic.

Corresponding author:

RNDr. Tomas Jecmen, Ph.D.

Tel.: +420 221 951 281.

E-mail address: jecmen@natur.cuni.cz

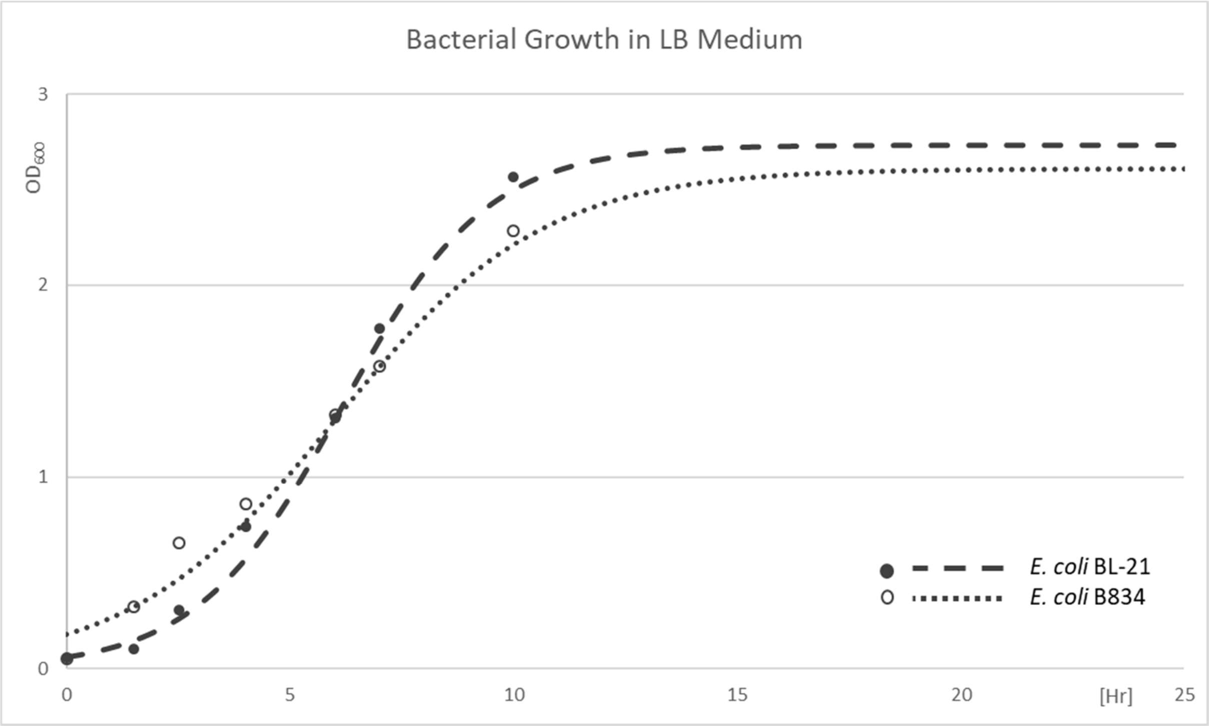

Figure S1. Bacterial growth of *E. coli* BL-21 and *E. coli* B834 in LB medium.
OD_{600} – optical density.

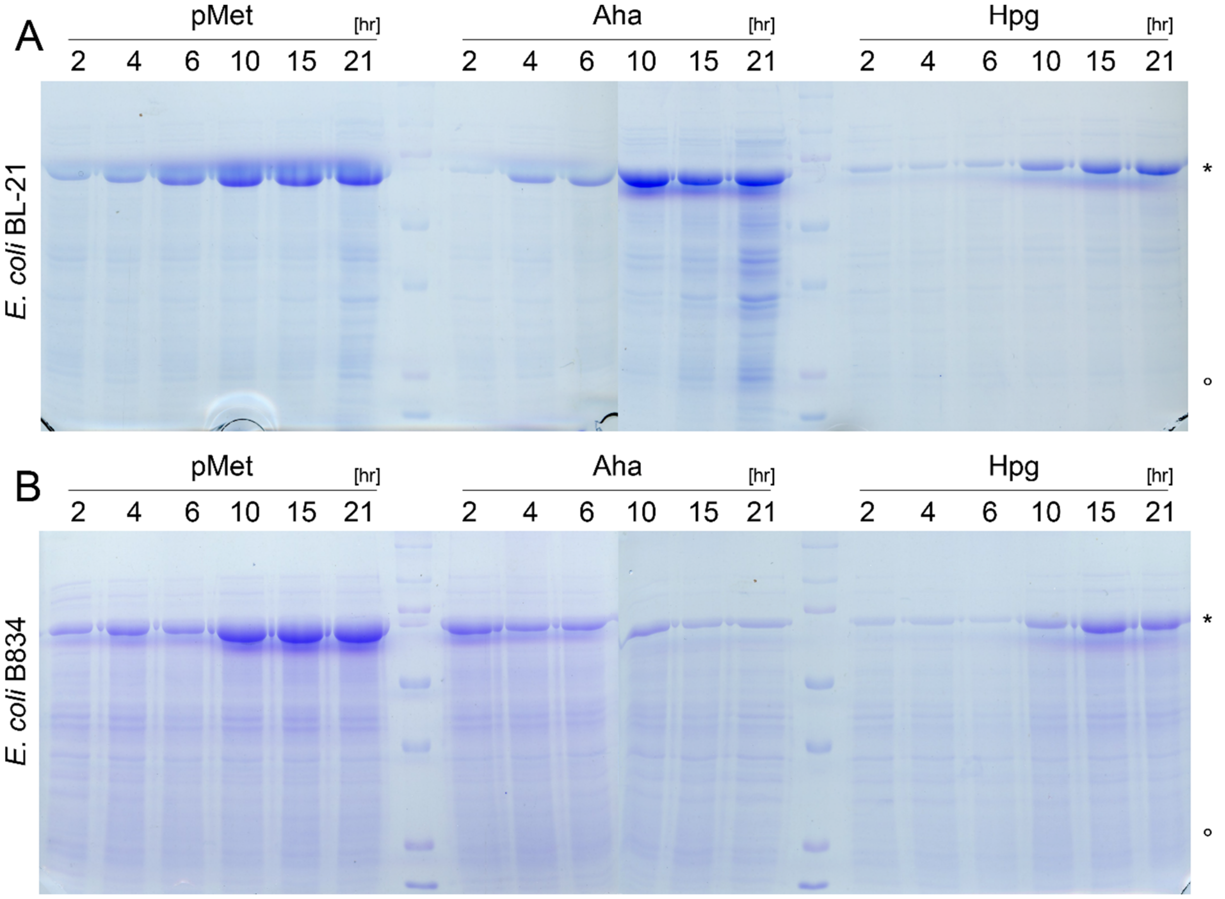

Figure S2. MBP-GFP expression levels in the presence of ncAAs. Bacterial suspension collected during cultivation at indicated time points separated on 10% SDS-PAGE and stained by Coomassie Brilliant Blue R250 stain. Legend: Expression in *E. coli* BL-21 (A) and *E. coli* B834 (B) in the presence of pMet (left), Aha (middle), and Hpg (right); Marked proteins: * MBP-GFP, ° pspA.

Figure S3

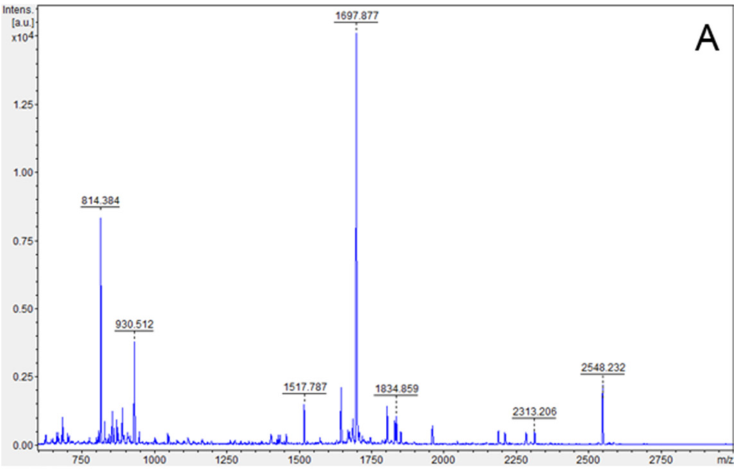

MATRIX SCIENCE **MASCOT Search Results** B

Mascot Score Histogram

Protein score is -10*Log(P), where P is the probability that the observed match is a random event.
Protein scores greater than 56 are significant (p<0.05).

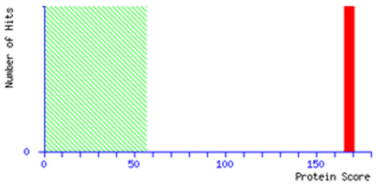

Protein View: PSPA_ECO57

Phage shock protein A OS=Escherichia coli O157:H7 OX=83334 GN=pspA PE=3 SV=2

**Database:**	SwissProt
**Score:**	168
**Expect:**	3.7e-13
**Monoisotopic mass (M_r):**	25477
**Calculated pI:**	5.39
**Taxonomy:**	Escherichia coli O157:H7

Sequence similarity is available as an NCBI BLAST search of PSPA_ECO57 against nr.

Search parameters

**Enzyme:**	Trypsin: cuts C-term side of KR unless next residue is P.
**Fixed modifications:**	Carbamidomethyl (C)
**Variable modifications:**	Oxidation (M)
**Mass values searched:**	9
**Mass values matched:**	9

Protein sequence coverage: 49%

Matched peptides shown in ***bold red***.

```
  1 MGIFSRFADI VNANINALLE KAEDPQKLVR LMIQEMEDTL VEVRSTSARA
 51 LAEKKQLTRR IEQASAREVE WQEKAELALL KEREDLARAA LIEKQKLTDL
101 IKSLEHEVTL VDDTLARMKK EIGELENKLS ETRARQQALM LRHQAANSSR
151 DVRRQLDSGK LDEAMARFES FERRIDQMEA EAESHSFGKQ KSLDDQFAEL
201 KADDAISEQL AQLKAKMKQD NQ
```

Unformatted sequence string: 222 residues (for pasting into other applications).

Sort by ◉ residue number ○ increasing mass ○ decreasing mass
Show ◉ matched peptides only ○ predicted peptides also

Start – End	Observed	Mr(expt)	Mr(calc)	ppm	M	Peptide
7 – 21	1644.8800	1643.8727	1643.8882	-9.43	0	R.FADIVNANINALLEK.A
7 – 27	2313.2060	2312.1987	2312.2012	-1.06	1	R.FADIVNANINALLEKAEDPQK.L
60 – 67	930.5120	929.5047	929.5042	0.52	1	R.RIEQASAR.E
103 – 117	1697.8770	1696.8697	1696.8632	3.86	0	K.SLEHEVTLVDDTLAR.M
121 – 133	1517.7870	1516.7797	1516.7733	4.27	1	K.EIGELENKLSETR.A
136 – 142	859.4740	858.4667	858.4745	-9.10	0	R.QQALMLR.H
168 – 173	814.3840	813.3767	813.3657	13.5	0	R.FESFER.R
174 – 189	1834.8590	1833.8517	1833.8315	11.0	1	R.RIDQMEAEAESHSFGK.Q
192 – 214	2548.2320	2547.2247	2547.2704	-17.9	1	K.SLDDQFAELKADDAISEQLAQLK.A

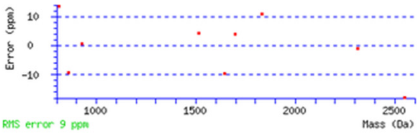

Figure S3: pspA protein identification by mass fingerprinting. A - MALDI-TOF spectrum with labeled m/z values used for database searches; B - Mascot search results.

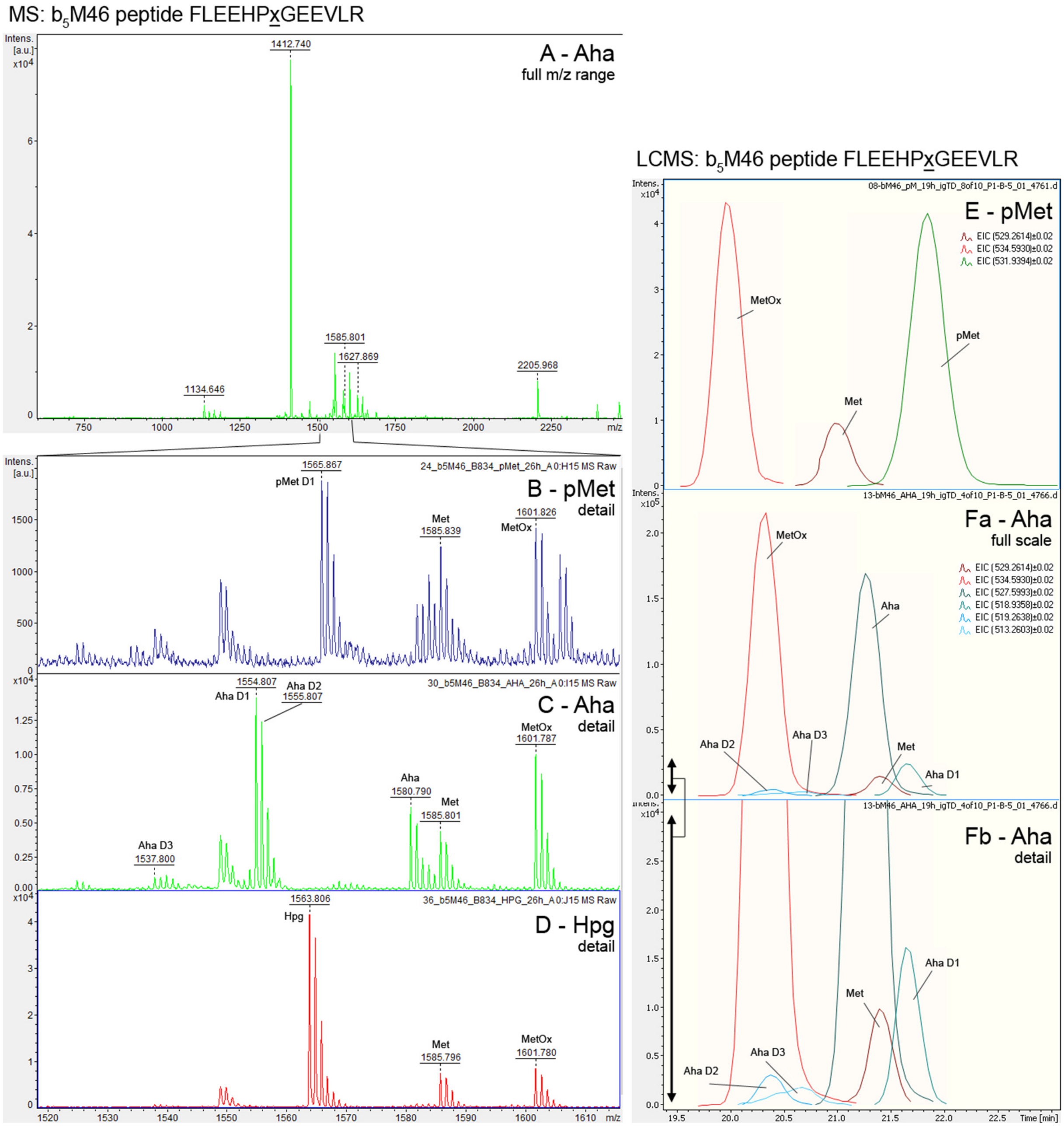

Figure S4: Comparison between MS and LC-MS data used for ncAA incorporation determination. Tryptic digest of b5M46 expressed for 26 hours in *E. coli* B834 with pMet (B,E), Aha (C,Fab), or Hpg (D); full m/z range MALDI-TOF spectrum of b5M46 (A) and spectra of FLEEHPxGEEVLR peptide with x site occupied by methionine, oxidized methionine, ncAA or it's derivative in detail (B-D); extracted ion chromatograms of the same peptide (E-Fab)

M9 Minimal Medium Recipe

M9 medium (1×) was prepared by mixing the stock solutions listed below and adding sterile water to the final volume prior each experiment. The stock solutions were stored for up to 2 months at 4 °C except for the 10× AAs, which was stored up to 1 week, and 50× nnAA, which was prepared fresh each time. The stock solutions were either autoclaved (^{A}) or filter sterilized (^{F}).

Stock solutions with the respective dilution factors:

- **M9 (5×):** 210.0mM Na_2HPO_4; 27.6 mM KH_2PO_4; 89.7 mM NH_4Cl; 42.8 mM NaCl; pH 7,4 ^{A}
- **Glucose (50×):** 20 % (w/v) Glucose ^{F}
- **$MgSO_4$ (200×):** 1 M $MgSO_4$ ^{A}
- **Vitamin B_1 (100×):** 1 % (w/v) Thiamin hydrochloride ^{F}
- **$CaCl_2$ (1000×):** 0.1 M $CaCl_2$ ^{A}
- **AAs (10×):** 0.5 mg/ml L-Isoleucine; 0.5 mg/ml L-Valine; 0.5 mg/ml L-Leucine; 1.0 mg/ml L-Lysine; 1.0 mg/ml L-Phenylalanine; 1.0 mg/ml L-Threonine ^{F}
- **Trace metals (1000×):** 80.6mM EDTA; 2.2mM $CoCl_2.6H_2O$; 92.5mM $FeSO_4.7H_2O$; 13.5mM $CuSO_4.5H_2O$; 10.6mM $MnCl_2.4H_2O$, 1.6mM H_3BO_3 ^{A}
- **ATB (1000×):** 100 mg/ml Ampicillin ^{F}
- **nnAA (50×):** 50 mM nnAA ^{F}

qTOF MS Acquisition Method Parameters

- Ion Polarity: Positive
- Mass Range: 150 to 2200 m/z
- Spectra Rate: 1 Hz

1/ Mode

- Save Spectra: Line Spectra Only
- Absolute Threshold: 100 cts.
- Peak Summation Width: 5 pts.

2/ Source

- End Plate Offset: 500 V
- Capillary: 4500 V
- Nebulizer: 2.0 Bar
- Dry Gas: 12.0 l/min
- Dry Temp: 180 °C

3/ Tune

- Transfer
  - Funnel 1 RF: 400.0 Vpp
  - isCID Energy: 0.0 eV
  - Multipole RF: 400.0 Vpp
- Quadrupole
  - Ion Energy: 3.5 eV
  - Low Mass: 300.0 m/z
- Collision Cell
  - Collision Energy: 6.0 eV
  - Collision RF: 1200 Vpp
  - Transfer Time: 90.0 µs
  - Pre Pulse Storage: 10.0 µs

4/ MS/MS

<u>Auto MS/MS</u>

- Cycle Time: 3.0 sec
- Absolute Threshold: 5000 cts.
- Active Exclusion
  - Exclude after: 1 Spectra
  - Release after: 0.20 min
  - Reconsider Precursor, if Current Intens. / Previous Intens.: 3.0

<u>Preferences</u>

- Charge State
  - Preferred Range: 2 – 5
  - Exclude Unknown
  - Group Length: 5

CID

- Isolation + Fragmentation List

Type	Mass	Width	Collision	Charge State
Base	300.0000	3.00	34.00	1
Base	500.0000	4.80	39.00	1
Base	1000.0000	6.00	52.00	1
Base	2000.0000	9.00	55.00	1
Base	300.0000	3.00	26.00	2
Base	500.0000	4.80	34.00	2
Base	1000.0000	6.00	40.00	2
Base	2000.0000	9.00	45.00	2
Base	300.0000	3.00	21.00	3
Base	500.0000	4.80	28.00	3
Base	1000.0000	6.00	36.00	3
Base	2000.0000	9.00	40.00	3

Acquisition

- MS/MS
  - Low: 10000 cts, 1 Hz
  - High: 50000 cts, 2 Hz
